# Train the Trainer: Hematopoietic Stem Cell Control of Trained Immunity

**DOI:** 10.3389/fimmu.2022.827250

**Published:** 2022-01-27

**Authors:** Marco De Zuani, Jan Frič

**Affiliations:** ^1^ International Clinical Research Center, St. Anne’s University Hospital, Brno, Czechia; ^2^ Institute of Hematology and Blood Transfusion, Prague, Czechia

**Keywords:** trained immunity, hematopoietic stem cells, HSPCs, innate immunity, myeloid cells, progenitor cells

## Abstract

Recent evidence shows that innate immune cells, in addition to B and T cells, can retain immunological memory of their encounters and afford long-term resistance against infections in a process known as ‘trained immunity’. However, the duration of the unspecific protection observed *in vivo* is poorly compatible with the average lifespan of innate immune cells, suggesting the involvement of long-lived cells. Accordingly, recent studies demonstrate that hematopoietic stem and progenitor cells (HSPCs) lay at the foundation of trained immunity, retaining immunological memory of infections and giving rise to a “trained” myeloid progeny for a long time. In this review, we discuss the research demonstrating the involvement of HSPCs in the onset of long-lasting trained immunity. We highlight the roles of specific cytokines and Toll-like receptor ligands in influencing HSPC memory phenotypes and the molecular mechanisms underlying trained immunity HSPCs. Finally, we discuss the potential benefits and drawbacks of the long-lasting trained immune responses, and describe the challenges that the field is facing.

## Introduction

Traditionally, the immune system of vertebrates has been binary classified into the innate and adaptive arms of immunity. While cells of the innate arm promptly recognize and respond to infections through pathogen recognition receptors (PRRs), cells of the adaptive arm rely on the somatic recombination of genes encoding immunoglobulin domains to recognise non-self molecules (1). Although immunological studies on plants and invertebrates – both lacking adaptive immune responses – have suggested that these organisms can become increasingly resistant to reinfections, it was long believed that only B and T cells can confer immunological memory to their host (2–4).

In the last 10 years, a growing body of evidence has demonstrated that innate immune cells can also develop immunological memory in vertebrates – a phenomenon that is termed ‘trained immunity’ or ‘innate immune memory’ (5). In vertebrates, certain cells have been found to develop trained immunity, i.e., monocytes (6), dendritic cells (7), neutrophils (8), and innate lymphoid cells such as NK cells (9) and ILC1s (10). This process mainly relies on the reprogramming of cellular metabolic pathways and the alteration of epigenetic markers regulating the expression of different sets of genes (6, 11–13). While trained immunity generally refers to the induction of an hyperinflammatory phenotype, it was demonstrated that also the generation of opposite phenotypes, such as in the case of ‘endotoxin tolerance’, relies on similar mechanisms (12, 14, 15). Although the molecular basis of trained immunity has mostly been described *in vitro* using human monocytes and *in vivo* using murine models of infection, a few studies have demonstrated that the same mechanisms also apply *in vivo* to humans (16–18). Similarly, epidemiological studies proved that whole-organism vaccinations (such as Bacillus Calmette-Guerin [BCG], smallpox, and measles vaccines) afford unspecific protection against infections not specifically targeted by the vaccines, resulting in a reduction in all-cause mortality (19–22). Recent studies demonstrated that long-lived, self-renewing cells are involved in the onset of trained immunity and, indeed, participate in long-lasting protection against pathogens. For example, alveolar macrophages are able to replenish populations of tissue-resident macrophages in the lungs independently of bone marrow (BM)-derived monocytes and were shown to offer cross-protection against lung pathogens after an initial intranasal challenge with *Acinetobacter baumannii* (23, 24). However, the duration and extent of the unspecific peripheral protection *in vivo* (which is measured in years) is poorly compatible with the average lifespan of human monocytes (5 to 7 days), suggesting that other long-lived cells, such as hematopoietic stem and progenitor cells (HSPCs, with lifespans ranging from 10 to 60 months), might be involved in this phenomenon (25–27). In agreement with this hypothesis, several studies published in the last few years suggest that specific signals carried by HSPCs are able to induce trained immunity in these cells, which, in turn, gives rise to a pool of ‘trained’ myeloid progeny.

Here, we present the latest advancements in the field of trained immunity, describe the known mechanisms of trained immunity in HSPCs, and discuss the challenges and remaining controversies that the field is facing.

## Induction of Trained Immunity in HSPCs

During infection and inflammation, immune effector cells are rapidly consumed at the sites of infection. As a result, HSPCs need to adapt their proliferation and differentiation rates to meet the increased demand of blood cells. To this end, HSPCs respond to different signals coming either from the BM niche, from distal inflamed sites, or from the infectious agents themselves (28). HSPCs have long been known to respond to multiple cytokines; however, several studies have demonstrated that both murine and human HSPCs express different Toll-like receptors (TLRs), and that direct engagement of these receptors with their ligands can alter the cell differentiation programming and, thus, their cellular output (28–32). HSPCs can also egress from the BM and migrate to tissues, where they can give rise to local populations of immune cells – a process known as ‘extramedullary haematopoiesis’ (33). Interestingly, different studies have reported that HSPCs can home back to the BM after migrating to peripheral tissues, increasing their chance of encountering TLR ligands and building a long-term memory of these encounters (34, 35).

In recent years, several studies have demonstrated that both human and murine HSPC compartments are at the foundation of the long-lasting ‘peripheral’ trained immunity observed *in vivo* (17). As we will discuss in the next chapters, some of these reports suggest that direct signaling from cytokines and microbial ligands to HSPCs play a central role in the reprogramming of HSPCs and their progeny (**Table 1**).

**Table 1:** Experimental studies demonstrating the direct involvement of HSPCs in the induction of innate immune memory.

Mediator	Model	Effect on HSPCs	Mouse	Human	Refs
IL-1β	β-glucan i.p.	Increased BM GMP frequency, reduction of MPP4 frequency; β-glucan exposed LT-HSCs give rise to more Gr1^+^ CD11b^+^ cells, and less CD19^+^ progeny; Improved HSPCs chemoresistance; Increased expression of genes involved in myeloid-skewing, immune functions and metabolic pathways (glycolysis, mevalonate) in LT-HSCs; Increased glycolysis in ckit+ progenitors and promotes alterations in cholesterol biosynthesis pathway; Phenotype abolished in mice treated with IL1Ra.	X		(36)
IL-1	β-glucan i.p.	Increased BM LSK cells counts - particularly LT-HSCs, MPPs and GMPs; Phenotype abolished in *Il1r^-/-^* mice and WT mice receiving IL1Ra.	X		(37)
IFN-γ	BCG vaccination (i.v. and s.c.)	i.v. but not s.c. increased ST-HSC and MPP counts in BM BCG i.v. induced the expression of genes involved in cell cycle and skew to myeloid MMP3 over MMP4 progenitors; BCG i.v. Induced IFN-γ gene signature in HSCs; BCG-exposed HSC afforded protection against Mtb infection; Phenotype reverted in *IfnγR^-/-^* mice.	X		(38)
IFNα, IFNβ	Mtb and BCG infection (i.v. and aerosol)	BCG and Mtb increased BM LSK cells numbers and the frequency of Ki67^+^ LSKs, ST-HSCs and MPPs; Mtb reduced the frequency of CMPs and GMPs, increases CLPs frequency; Mtb induced RIP3K-dependent necroptosis of CMPs and GMPs; Mtb increased Stat1 expression in all HSPCs clusters and induces type-I IFNs gene expression; Mtb reduced HSPCs engraftment potential, BCG enhances it.	X		(27)
Leptin (indirect)	Voluntary exercise	Exercise reduced LSK proliferation and lineage commitment; Decreased chromatin accessibility long after exercise; Metabolic switch from glycolysis (sedentary) to OXPHOS (exercise); Increased cellular output in exercised mice after LPS injection.	X		(39)
HNF1A/B (TF)	BCG intradermal vaccination	Unaltered counts of BM progenitor counts; Increased granulocytic/myeloid lineage transcripts in HSPCs 90 days post-vaccination; Increased predicted activity of HNF1A/B TF.		X	(17)
Dectin-1	Depleted zymosan and *Candida albicans* i.v.	Increased HSPCs proliferation in response to direct dectin1 ligation by depleted zymosan; Increased output of CD11b+ F4/80++ cells with heightened *ex-vivo* production of inflammatory cytokines; Phenotype reverted in *dectin1^-/-^* and *Myd88^-/-^* HSPCs.	X		(40)
TLR4 - C/EBPα (TF)	LPS i.p.	Rapid and transient increase of LT-HSC, MMP2, MMP3 and GMP numbers, reduction of MPP4 numbers; Stable change in Flt3L^-^ LSK chromatin accessibility 12 weeks after LPS; Increased myeloid differentiation, and OXPHOS and fatty acid metabolism gene signature during secondary LPS challenge; Phenotype strongly reverted in *Tlr4^-/-^* HSPCs, completely abolished in C/EBPβ HSPCs.	X		(41)
Heme	Heme i.p.	Decrease of BM LT-HSC and committed progenitors; Long-term (28 days) changes in LSK chromatin accessibility, especially affecting binding sites for stemness-associated TFs (Runx, Nfi, Spi); Open chromatin peaks associated to genes promoting myeloid cell differentiation and restraining megakaryocyte differentiation.	X		(42)
NLRP3	Western diet	Increased abundance of MPPs and GMPs; Transcriptional reprogramming of GMPs promoting cell proliferation and monocytic development, and restraining granulocytic development; Long lasting upregulation of IFN response genes and inflammatory signaling in response to LPS challenge, compared to normal diet; Long-lasting changes in GMP accessible chromatin landscape; Phenotype reverted in *Nlrp3^-/-^* mice.	X		(43)

The table summarizes the response of HSPCs during the induction of innate immune memory. Each of these studies also demonstrated that the direct stimulation on HSPCs was responsible for the induction of long-lasting, specific memory phenotypes on myeloid cells. i.p., intra-peritoneal injection; i.v., intravenous; s.c., sub-cutaneous; BCG, Bacillus Calmette-Guérin; BM, bone marrow; CLP, common lymphoid progenitor; CMP, common myeloid progenitor; GMP, granulocyte-monocyte progenitor; HSC, hematopoietic stem cell, HSPC, hematopoietic stem and progenitor cell, IL1Ra, IL-1 receptor antagonist; LPS, lipopolysaccharide; LSK, Lin- Sca1+ Kit+ cells; LT-HSC, long term-HSC, MPP, multipotent progenitor; Mtb, Mycobacterium tuberculosis; OXPHOS, oxidative phosphorylation; ST-HSC, short term HSC; TF, transcription factor.

### Cytokine Signaling on HSPCs Promote Trained Immunity

Cytokines and growth factors play crucial roles in haematopoiesis by regulating HSPC quiescence, proliferation and differentiation (44). Similarly, signaling from many cytokines, such as G-CSF, GM-CSF, M-CSF, IL-6, IL-1β, and Type-I and II IFNs, control the transition to emergency haematopoiesis, i.e., the adaptation of the hematopoietic process during infection (28).

Two recent studies demonstrated that stimulation with β-glucan (a fungal cell wall component known to be a strong inducer of trained immunity in monocytes) also induces long-term trained immunity in murine HSPCs and that IL-1β plays a key role in HSPC reprogramming (6, 36, 37). Mitroulis and colleagues showed that, in a murine model, the intraperitoneal injection of β-glucan acted on CD41^+^ myeloid-skewed long-term hematopoietic stem cells (LT-HSCs), resulting in the expansion of granulocyte-monocyte progenitors (GMPs) and the reduction of lymphoid-skewed multipotent progenitors (MPP4). These myeloid skews in the HSPCs populations were accompanied by the profound metabolic reprogramming of cKit^+^ progenitors, which showed increased glycolytic activity and alterations in their cholesterol biosynthesis pathway. Compared to PBS-treated animals, mice primed with β-glucan showed a protective response to a later lipopolysaccharide (LPS) injection, as demonstrated by the enhanced expansion of multipotent progenitors (MPPs) and Lin- Sca1+ cKit+ (LSK) cells in the BM and by the mitigation of DNA damage at the level of LT-HSCs. Interestingly, the authors found that IL-1β signaling was required for the metabolic reprogramming and lineage skewing of LT-HSCs following β-glucan administration, as the pharmacological inhibition of IL-1 by the IL-1 receptor antagonist Anakinra completely abrogated these effects (36). Similarly, in a study by Moorlag and colleagues, β-glucan administration caused the expansion of LT-HSCs, MPPs and GMPs, which was abolished in IL-1R^−/−^ mice as well as wildtype (WT) mice that received Anakinra. Priming with β-glucan also protected the mice against pulmonary infection with virulent *Mycobacterium tuberculosis* (Mtb), possibly as a result of the enhanced expression of antimicrobial cytokines by myeloid cells. Again, the protective effect provided by β-glucan was completely abolished in IL-1R^−/−^ mice and in Anakinra-treated WT mice, suggesting that IL-1 plays a key role in the induction of protective trained immunity in HSPCs (37).

IL-1 was also found to have a prominent role in the induction of innate immune reprogramming following a western diet regime. This study employed the *Ldlr^-/-^* arteriosclerosis mouse model and showed that, when fed a western diet, these animals developed a systemic inflammatory response and long-lasting hyper-responsiveness of myeloid cells that continued after switching back to a chow diet. Mechanistically, the western diet induced the persistent transcriptional and epigenetic reprogramming of BM GMPs, which was mediated by NLRP3 activation and the subsequent release of IL-1. Accordingly, this phenotype was largely blunted in *Nlrp3^-/-^ Ldlr^-/-^* mice as well as in animals receiving a recombinant IL-1ra (43).

Type-I and II interferons (IFNs) were also reported to play a key role in the induction of protective or detrimental innate immune memory in murine HSPCs. Two recent studies employed different models of BCG or Mtb infection in mice to determine their effect on the hematopoietic compartment (27, 38). Interestingly, both studies demonstrated that, to affect HSPC biology, the bacteria had to reach the BM, although LSK cells were not directly infected, suggesting an indirect effect was involved. Kaufman and colleagues showed that HSCs isolated from mice exposed to BCG were myeloid-skewed and able to generate an epigenetically reprogrammed myeloid progeny which protected against Mtb infection *in vivo* (38). The authors suggested that INF-γ signaling by HSCs was required for the generation of this protective response, as mice lacking the IFN-γ receptor failed to generate macrophages with a protective phenotype. Similarly, Khan et al. compared the responses of the hematopoietic compartment to either BCG or virulent Mtb infection (27). They found that Mtb, but not BCG, reduced myelopoiesis by inducing the Ripk3-dependent necroptosis of common myeloid progenitors (CMPs) and GMPs, and impaired HSC engraftment potential. Compared to BCG-exposed HSCs, cells exposed to Mtb showed an upregulation of Stat1 and type-I IFN targets, thus suggesting the involvement of IFN-α or IFN-β in this process. Concordantly, mice lacking the type-I IFN receptor Ifnar1 were afforded more protection against Mtb infection comparted with WT animals. Similarly, the systemic administration of poly(I:C) – a strong inducer of type-I IFNs – recapitulated the negative impact on myelopoiesis observed in Mtb-infected animals, which was completely reverted in Ifnar1^-/-^ mice. Taken together, these studies suggest that the balance between type-I and type-II IFN signaling by HSPCs plays a key role in the induction of a detrimental or protective innate immune memory *in vivo*. However, different studies demonstrated that chronic exposure of murine and human HSCs to IFN-γ impairs their maintenance and self-renewal, indicating that the route of infection and the duration of the stimulus play key roles in the regulation of HSC fate (45, 46).

Finally, using a murine model of voluntary exercise, Frodermann and colleagues showed that, by diminishing plasma levels of the hormone leptin, exercise reduced HSPC proliferation and lineage commitment, resulting in fewer circulating leukocytes (39). In particular, reduced leptin signaling resulted in the increased production of ‘quiescence-inducing factors’ by LepR^+^ stromal cells which, in turn, reduced the LSK chromatin accessibility of multiple genes involved in proliferation and myelopoiesis for several weeks after exercise interruption. Surprisingly, compared to sedentary mice, exercised mice were more fully protected against LPS injection and caecal ligation and puncture, as highlighted by their lower mortality and increased cellular output. Although the authors failed to identify the nature of these quiescence-inducing factors, it is likely that cytokines such as SCF are involved in the reprogramming of HSCs in the BM niche (47, 48).

### Direct TLR Ligation Promotes Trained Immunity in the HSPC Pool

In the last two decades, several studies have demonstrated that both human and murine HSPCs express many PRRs (e.g., TLR-1, -2, -3, -4, -6, -7, -8, NOD2) and are able to respond to their engagement by activating specific downstream signaling pathways (29, 49, 50). TLR ligation on HSPCs, in particular, promotes their exit from quiescence and proliferation and results in a differentiation skew that favours a myeloid over a lymphoid cell fate (31, 32, 49, 50). Moreover, stimulation with TLR ligands induces cytokine secretion in virtually all HSPC subsets (50–52).

More recently, reports suggest that HSPCs stimulated with various TLR-ligands show memory-like traits, particularly those affecting the functionality of their myeloid progeny. For example, several studies from the Gil and Goodridge labs showed that *in vitro* differentiation of murine Lin-HSPCs exposed to TLR2 and TLR4 ligands (either *in vitro* or *in vivo*) generate macrophages with a decreased ability to release proinflammatory cytokines and reactive oxygen species in response to TLR ligation compared to control cells. However, HSPCs differentiated in the presence of *Candida albicans* yeast generated macrophages with a heightened production of inflammatory cytokines and improved fungicidal activity (52–55). Although they outlined a strong rationale for the involvement of HSPCs in building systemic trained immunity, these studies failed to determine the duration of these memory traits in HSPCs or conclude whether TLR ligation, rather than the production of autocrine soluble factors, is uniquely responsible for the observed phenotypes. A recent study, however, showed that the latter is likely to be the case: while direct TLR2 ligation on Lin-cells was required to promote myeloid differentiation, the differential response of macrophages derived from cells exposed to TLR2 and dectin-1 ligands was completely dependent on HSPC secretome release in response to these ligands (40). Interestingly, as LT-HSC do not express dectin-1, it is possible that myeloid-committed progenitors expressing dectin-1 are responsible for the secretion of training cytokines and factors (56).

However, it was recently reported that the exposure of HSPCs to LPS is sufficient to induce long-lasting innate memory in murine HSPCs (41). Using *in vivo* models of mixed BM chimaera and LSK cell transplantation, the authors showed that LPS-exposed LT-HSCs provide long-term protection against *Pseudomonas aeruginosa* infection for more than 12 weeks. This phenomenon relied on the persistent epigenetic reprogramming of HSCs, which showed increased chromatin accessibility, especially in myeloid enhancers, which were later re-activated during reinfection. Interestingly, direct signaling through TLR4 was fundamental to the induction of immune memory in HSCs, as TLR4^−/−^ HSCs almost completely lost the epigenetic signature induced by LPS exposure in the TLR4^−/−^:WT BM cell chimaera. Furthermore, the authors found that, while not necessary for inducing the initial response to LPS by HSCs, C/EBPβ transcription factor (TF) was required to maintain the long-term accessibility of regulatory elements controlling the expression of myeloid and inflammatory genes during re-infection (41).

C/EBPβ and C/EBPα were also found to play key roles in the differentiation of human CD34+ cells towards the myeloid lineage in response to LPS *in vitro*. In this case, however, LPS activated a TLR4-MD2-MyD88 pathway that resulted in the expression of IL-6, which in turn, induced C/EBPα and C/EBPβ phosphorylation, finally promoting monocytic-macrophage differentiation (57). Direct TLR4 triggering of HSCs also plays an important role in the maintenance of HSC fitness. Research showed that the TLR4-MyD88 pathway in HSPCs seems to regulate myeloid suppression, while the TLR4-TRIF pathway plays a key role in regulating HSC proliferation (30, 58, 59). Concordantly, *in vivo* LPS and *Salmonella typhimurium* infection resulted in the strong proliferation of LT-HSCs through a TRIF-ROS-p38 pathway, which resulted in proliferative stress, DNA damage, and the long-term impairment of their repopulating ability (30).

Among non-canonical TLR4 ligands, heme was recently described to induce trained immunity in human monocytes as well as murine HSPCs (42, 60, 61). In fact, a single injection of heme protected a mouse model of polymicrobial sepsis from death when challenged 7 days post heme-administration. When sepsis was induced 28 days after the initial heme training, this protection completely vanished. A further analysis of BM HSPCs 28 days after heme training revealed that chromatin accessibility in the ST-HSC and MPP3 subsets was substantially different than that in control mice. These differences further reflected the alteration in the chromatin accessibility to TFs that are important for the maintenance of stemness and the promotion of myelopoiesis (e.g., Gata2, Runx1, Nfi) (42).

Finally, it was recently revealed that young and aged LT-HSCs display different memory traits after TLR activation. In aged LT-HSCs, but not in young cells, brief *ex vivo* exposure to TLR2 and TLR4 ligands induced the expansion of peripheral myeloid cells and a reduction in lymphoid cells 3 months after transplantation. This was possibly the result of the initial expansion of a CD61+ subset of LT-HSCs in the aged mice, which were found to be more proliferative and intrinsically prone to a myeloid output (62).

Taken together, these studies suggest that direct TLR ligation on HSPCs plays an important role in the terminal differentiation of the cells as well as the establishment of innate immune memory. Interestingly, both direct TLR signaling and the indirect signaling autocrinally induced by the production of cytokines seem to be equally involved in this complex phenomenon.

## Discussion

A growing body of evidence, examples of which are presented in this review, highlights the key roles of HSPCs in maintaining the long-lasting aspects of trained immunity. However, several questions remain to be addressed regarding the conundrum of trained immunity and its implications.

### Diamonds Are Forever, Trained Immunity Is Not

Although most of the studies support the idea that trained immunity phenotypes are retained for a very long time, a key remaining aspect of trained immunity that still has to be clearly defined, is the specific duration of such phenotype *in vivo* in humans. This, as we will discuss later, has important implications on the possible translatability of such evidence to human health. Studies using murine models showed that BM HSPCs can retain an epigenetic memory, affecting the response of their myeloid progeny for over 1 year (27). Similarly, epidemiological studies suggest that BCG vaccination might afford unspecific protection for up to 5 years (63). Although it is still a matter of discussion and an object of ongoing clinical trials, recent epidemiological studies that aimed to assess the protective effects of BCG vaccination against SARS-CoV-2 infections showed that a history of BCG vaccination was associated with a decreased rate of infection and an overall improvement in the symptomatology (64, 65). Furthermore, a recent study suggested that HSCs can establish and retain a persistent epigenetic memory (characterised by drastic differences in DNA methylation and chromatin accessibility) imposed during their development, which eventually direct HSC functions during exposure to stressors (66). It is, therefore, likely that the duration of such an innate immune memory in HSCs can be measured in the context of years and that the additional effects of subsequent stresses and infections on such cells are maintained lifelong. Finally, a breakthrough study recently demonstrated that unspecific protection against bacterial infections afforded by a sublethal infection with *C. albicans* was transmitted intergenerationally and transgenerationally for up to two generations (67). Strikingly, the progeny of *C. albicans*-exposed mice inherited an epigenetic signature induced by the fungal insult from the HSPC populations of their parents, resulting in improved myeloid cell output and activation and increased survival following *E. coli* infection. These results are in agreement with a randomized controlled trial showing that the reduction in mortality observed in BCG-vaccinated infants was significantly improved if the mother also had a history of BGC vaccination (68). Understanding the specific duration of trained immunity induced by different agents (e.g. BCG, β-glucans) will be fundamental to design effective therapies.

### All That Glitters Is Not Gold: Trained Immunity as a Double-Edged Sword in Inflammaging and Chronic Inflammation

Most of the studies performed so far have demonstrated that the typical enhancement of myeloid cell activation afforded by trained immunity leads to the cells having a protective role against unspecific infections, however, less is known about the putative side effects that proinflammatory poising of the myeloid compartment might have in other scenarios (69). The proinflammatory poising of myeloid cells can undoubtedly be useful during infection; nevertheless, there is evidence that it can contribute to inflammatory diseases. For example, NLRP3 activation and IL-1 release during a western diet are known to cause the reprogramming of BM GMPs, resulting in proinflammatory poising of the myeloid compartment (43). Chronic exposure to IL-1 negatively affects the HSC pool, reducing their self-renewing capacity and restricting their lineage output (70). On a similar note, a single LPS injection was found to induce a trained phenotype of murine microglia; this in turn, promoted the neuropathology in a model of Alzheimer’s disease, increasing both amyloid-β levels and the plaque load, and augmented IL-1β levels after brain ischaemia (71).

**Figure 1:**
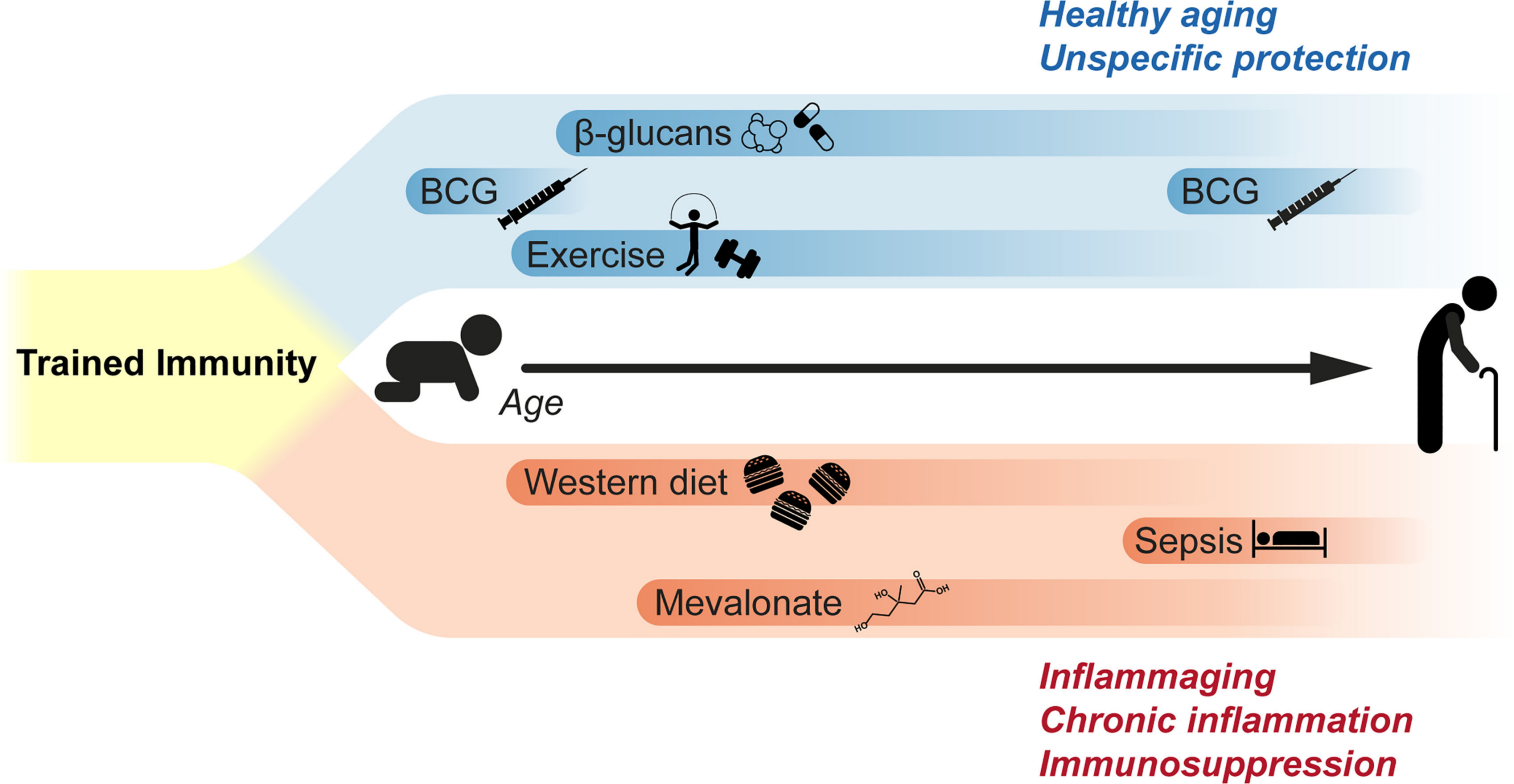
Impact of trained immunity during lifetime. Current studies suggest that trained immunity can have both beneficial and detrimental effects. β-glucan administration is known to induce a long-lasting reprograming of myeloid cells that results in the unspecific protection against different pathogens. BCG vaccination in children is known to afford protection and reduce all-cause mortality; similarly, recent clinical trials suggest that BCG vaccination in elderly can reduce the plasma levels of inflammatory cytokines and mediators, and to afford unspecific protection against respiratory infections. Voluntary exercise also induces analogous effects, decreasing the proliferation and lineage commitment of HSPCs while affording a long-term protection during sepsis. On the other side, western diet was found to reprogram bone marrow granulocyte progenitors, increasing the reactivity of innate immune cells. Similarly, signaling by metabolic intermediates as mevalonate was suggested to be involved in the pathological hyperinflammation affecting patients suffering from hyper-IgD syndrome. Finally, sepsis is also known to induce a long-term immunosuppression in survivors, possibly through mechanisms similar to those inducing trained immunity in hematopoietic progenitors and myeloid cells.

The hypothesis of a detrimental trained immunity response is also supported by a study on patients who were affected by hyper-IgD syndrome (HIDS), in which recurring attacks of sterile inflammation occur. Compared to healthy donors, the monocytes from these patients showed a trained immunity phenotype characterised by the enhanced production of inflammatory cytokines (both at the basal level and during infection) as well as an H3K27ac signature partially overlapping with that of *in vitro* trained monocytes. The authors speculated that this phenotype could be induced by the accumulation of mevalonate during HIDS which, in turn, activates the AKT-mTOR pathway (possibly through IGF1-R) and finally amplifies the molecular cascade that controls the epigenetic rewiring of trained monocytes (13). Similar observations linking the proinflammatory phenotype of monocytes to a characteristic epigenetic signature were made on patients affected by symptomatic arteriosclerosis (72) and coronary artery disease (73). Taken together, these studies point to a possible detrimental role of trained immunity, where monocytes (and possibly other myeloid cells) participate in the maintenance and worsening of inflammatory pathologies (**Figure 1**).

This gains even greater importance in the light of the long-lasting effect exerted on the HSPCs pool. It is, therefore, paramount to fully explore and identify these potential side effects before applying any treatment in the clinical practice. As a matter of fact, the pharmacological control of trained immunity is gaining more and more interest from the scientific community as a tool to boost the immunity of frail patients (74). Two recent clinical trials showed the promising effects of BCG re-vaccination in the elderly (75, 76). However, other researchers have speculated that continuous triggering of the immune system throughout life and, thus, the constant induction of trained immunity on HSPCs, could be the missing link between aging and the persistent low-grade inflammation observed in the elderly that is known as ‘inflammaging’ (77, 78). Therefore, dissecting the molecular mechanisms driving beneficial or detrimental trained immunity in HSPCs will be a fundamental step in the development of novel pharmacological strategies or towards the repurposing of already approved therapies. The induction of trained immunity can be achieved through stimulation with different PRR-ligands (especially ligands for dectin-1 and NOD2), while inhibition of trained immunity can be accomplished by interfering with metabolic pathways or epigenetic regulators (79). As such, great hope is placed on the use of small molecules as regulating epigenetic modifiers (80).

## Conclusion

Taken together, the studies presented in this review clearly put the HSPC compartment at the foundation of the long-term effects exerted by trained immunity. Direct induction of trained immunity in HSPCs results in the production of a myeloid progeny with a trained phenotype directly inherited from their progenitors. Although different studies point toward different factors, direct signaling by both cytokines (as IFNs and IL-1) and PRR ligands (as LPS and β-glucans) is fundamental for the induction of the metabolic and epigenetic rewiring in HSPCs responsible for the induction of trained immunity in HSPCs.

As the pharmaceutical control of trained immunity is gaining more and more interest by the scientific community, some aspects of this phenomenon still require thorough investigation in order to design effective therapeutic strategies. In particular, several studies report that trained immunity might participate in the onset and worsening of inflammatory pathologies such as arteriosclerosis. Knowing the derailments that can occur during the induction of trained immunity represent a key step for the development of safe treatments. Secondly, it is still not known the exact and specific duration of trained immunity induced by different factors. This would allow the design of effective ‘training therapies’ aimed to boost the innate response against infections without promoting an undesirable hyperinflammatory bias of the immune system which can lead to the development (or worsening) of inflammatory diseases. Finally, unravelling the common molecular pathways involved in the onset of trained immunity will allow the precise control of the balance between unspecific protection and undesirable hyperinflammation.

We believe that trained immunity represents a unique phenomenon that can be harnesser to design game changing-therapies: the direction is clear and the path paved.

## Author Contributions

MZ conceived and wrote the manuscript, and secured funding. JF supervised the writing and reviewed the manuscript, and secured funding. Both authors read and approved the final manuscript.

## Funding

This work was supported by the Ministry of Health of the Czech Republic, grant no. NU21J-05-00056 and DRO (Institute of Haematology and Blood Transfusion—UHKT, 00023736), and by the European Social Fund and European Regional Development Fund—Project ENOCH (no. CZ.02.1.01/0.0/0.0/16_019/0000868). MZ was supported by the European Regional Development Fund—Project Support of MSCA IF fellowships at FNUSA-ICRC (no CZ.02.2.69/0.0/0.0/19_074/0016274).

## Conflict of Interest

The authors declare that the research was conducted in the absence of any commercial or financial relationships that could be construed as a potential conflict of interest.

## Publisher’s Note

All claims expressed in this article are solely those of the authors and do not necessarily represent those of their affiliated organizations, or those of the publisher, the editors and the reviewers. Any product that may be evaluated in this article, or claim that may be made by its manufacturer, is not guaranteed or endorsed by the publisher.
